# Human herpesvirus 6A and axonal injury before the clinical onset of multiple sclerosis

**DOI:** 10.1093/brain/awad374

**Published:** 2023-10-31

**Authors:** Viktor Grut, Martin Biström, Jonatan Salzer, Pernilla Stridh, Daniel Jons, Rasmus Gustafsson, Anna Fogdell-Hahn, Jesse Huang, Julia Butt, Anna Lindam, Lucia Alonso-Magdalena, Tomas Bergström, Ingrid Kockum, Tim Waterboer, Tomas Olsson, Henrik Zetterberg, Kaj Blennow, Oluf Andersen, Staffan Nilsson, Peter Sundström

**Affiliations:** Department of Clinical Science, Neurosciences, Umeå University, 901 87 Umeå, Sweden; Department of Clinical Science, Neurosciences, Umeå University, 901 87 Umeå, Sweden; Department of Clinical Science, Neurosciences, Umeå University, 901 87 Umeå, Sweden; Department of Clinical Neuroscience, Karolinska Institutet, 171 77 Stockholm, Sweden; Center for Molecular Medicine, Karolinska University Hospital, 171 76 Stockholm, Sweden; Department of Clinical Neuroscience, Institute of Neuroscience and Physiology, Sahlgrenska Academy, University of Gothenburg, 405 30 Gothenburg, Sweden; Department of Clinical Neuroscience, Karolinska Institutet, 171 77 Stockholm, Sweden; Center for Molecular Medicine, Karolinska University Hospital, 171 76 Stockholm, Sweden; Department of Clinical Neuroscience, Karolinska Institutet, 171 77 Stockholm, Sweden; Center for Molecular Medicine, Karolinska University Hospital, 171 76 Stockholm, Sweden; Department of Clinical Neuroscience, Karolinska Institutet, 171 77 Stockholm, Sweden; Center for Molecular Medicine, Karolinska University Hospital, 171 76 Stockholm, Sweden; Infections and Cancer Epidemiology Division, German Cancer Research Center, 69120 Heidelberg, Germany; Department of Public Health and Clinical Medicine, Unit of Research, Education and Development Östersund Hospital, Umeå University, 901 87 Umeå, Sweden; Department of Neurology, Skåne University Hospital and Department of Clinical Sciences, Lund University, 221 84 Lund, Sweden; Department of Infectious Diseases, Institute of Biomedicine, Sahlgrenska Academy, University of Gothenburg, 405 30 Gothenburg, Sweden; Department of Clinical Neuroscience, Karolinska Institutet, 171 77 Stockholm, Sweden; Center for Molecular Medicine, Karolinska University Hospital, 171 76 Stockholm, Sweden; Infections and Cancer Epidemiology Division, German Cancer Research Center, 69120 Heidelberg, Germany; Department of Clinical Neuroscience, Karolinska Institutet, 171 77 Stockholm, Sweden; Center for Molecular Medicine, Karolinska University Hospital, 171 76 Stockholm, Sweden; Department of Psychiatry and Neurochemistry, Institute of Neuroscience and Physiology, Sahlgrenska Academy, University of Gothenburg, 405 30 Gothenburg, Sweden; Clinical Neurochemistry Laboratory, Sahlgrenska University Hospital, 431 80 Mölndal, Sweden; Department of Neurodegenerative Disease, UCL Institute of Neurology, London, WC1N 3BG, UK; UK Dementia Research Institute at UCL, London, W1T 7NF, UK; Hong Kong Centre for Neurodegenerative Diseases, Hong Kong999077, China; Wisconsin Alzheimer’s Disease Research Center, University of Wisconsin School of Medicine and Public Health, University of Wisconsin-Madison, Madison, WI 53792, USA; Department of Psychiatry and Neurochemistry, Institute of Neuroscience and Physiology, Sahlgrenska Academy, University of Gothenburg, 405 30 Gothenburg, Sweden; Clinical Neurochemistry Laboratory, Sahlgrenska University Hospital, 431 80 Mölndal, Sweden; Department of Clinical Neuroscience, Institute of Neuroscience and Physiology, Sahlgrenska Academy, University of Gothenburg, 405 30 Gothenburg, Sweden; Department of Laboratory Medicine, Institute of Biomedicine, Sahlgrenska Academy, University of Gothenburg, 405 30 Gothenburg, Sweden; Department of Clinical Science, Neurosciences, Umeå University, 901 87 Umeå, Sweden

**Keywords:** multiple sclerosis, axonal injury, neurofilament light chain, human herpesvirus 6-A, Epstein-Barr virus

## Abstract

Recent research indicates that multiple sclerosis is preceded by a prodromal phase with elevated levels of serum neurofilament light chain (sNfL), a marker of axonal injury. The effect of environmental risk factors on the extent of axonal injury during this prodrome is unknown. Human herpesvirus 6A (HHV-6A) is associated with an increased risk of developing multiple sclerosis. The objective of this study was to determine if HHV-6A serostatus is associated with the level of sNfL in the multiple sclerosis prodrome, which would support a causative role of HHV-6A.

A nested case-control study was performed by crosslinking multiple sclerosis registries with Swedish biobanks. Individuals with biobank samples collected before the clinical onset of multiple sclerosis were included as cases. Controls without multiple sclerosis were randomly selected, matched for biobank, sex, sampling date and age. Serostatus of HHV-6A and Epstein-Barr virus was analysed with a bead-based multiplex assay. The concentration of sNfL was analysed with single molecule array technology. The association between HHV-6A serology and sNfL was assessed by stratified *t-*tests and linear regressions, adjusted for Epstein-Barr virus serostatus and sampling age. Within-pair ratios of HHV-6A seroreactivity and sNfL were calculated for each case and its matched control. To assess the temporal relationship between HHV-6A antibodies and sNfL, these ratios were plotted against the time to the clinical onset of multiple sclerosis and compared using locally estimated scatterplot smoothing regressions with 95% confidence intervals (CI).

Samples from 519 matched case-control pairs were included. In cases, seropositivity of HHV-6A was significantly associated with the level of sNfL (+11%, 95% CI 0.2–24%, *P* = 0.045) and most pronounced in the younger half of the cases (+24%, 95% CI 6–45%, *P* = 0.007). No such associations were observed among the controls. Increasing seroreactivity against HHV-6A was detectable before the rise of sNfL (significant within-pair ratios from 13.6 years versus 6.6 years before the clinical onset of multiple sclerosis).

In this study, we describe the association between HHV-6A antibodies and the degree of axonal injury in the multiple sclerosis prodrome. The findings indicate that elevated HHV-6A antibodies both precede and are associated with a higher degree of axonal injury, supporting the hypothesis that HHV-6A infection may contribute to multiple sclerosis development in a proportion of cases.

## Teaser

Grut *et al.* uncover an association between prior infection with human herpesvirus 6A (HHV-6A) and subtle axonal injury—as revealed by serum neurofilament light levels—in younger individuals who later develop multiple sclerosis. They conclude that HHV-6A infection may contribute to multiple sclerosis development in a proportion of cases.

See Cree (https://doi.org/10.1093/brain/awad418) for a scientific commentary on this article.

## Introduction

Multiple sclerosis is an immune-mediated chronic disease affecting the CNS.^1^ According to the prevailing hypothesis, the disease is triggered by an interplay of environmental risk factors in individuals with genetic susceptibility to multiple sclerosis.^2^ Virtually all patients with multiple sclerosis are seropositive for Epstein-Barr virus (EBV), which has thus been suggested as a prerequisite for the disease in adults.^3,4^ However, the high seroprevalence of EBV in the healthy population shows that EBV is not a sufficient cause of multiple sclerosis. Additional environmental factors are likely contributing to its aetiology. One such candidate is human herpesvirus 6 (HHV-6), which has been repeatedly associated with multiple sclerosis risk.^5^ While many previous studies did not distinguish between the two different species HHV-6A and -6B (collectively referred to as HHV-6), several studies now indicate that the association with multiple sclerosis is attributable to HHV-6A.^6-9^ As a neurotrophic virus, HHV-6A can infect oligodendrocytes.^10^ These cells produce myelin, often regarded as the target for the inflammatory processes in multiple sclerosis.^1^ In line with this finding, HHV-6 has been identified more frequently in biopsy samples from multiple sclerosis plaques than in normal CNS tissue.^11-13^ Higher levels of antibodies against HHV-6 have also been detected in serum and CSF from patients with multiple sclerosis.^7,14^ The level of these antibodies is associated with the risk of relapse in multiple sclerosis.^15^ In addition, higher levels of HHV-6 DNA have been observed in blood plasma from patients with multiple sclerosis.^16^ Interestingly, these signs of reactivated HHV-6 infection were only observed during relapses or exacerbations of multiple sclerosis.^16,17^

Previous research suggests that HHV-6 may be involved in the early stages of multiple sclerosis pathogenesis.^9,18^ The initiation and early phase of multiple sclerosis need to be better studied, but such investigations are complicated by the recently recognized prodrome.^19^ For example, two recent studies reported higher levels of serum neurofilament light chain (sNfL)—a marker of axonal injury—in biobank samples from individuals who several years later developed clinical signs of multiple sclerosis.^20,21^ These ultrasensitive analyses of sNfL have also been used to investigate the order of events in EBV seroconversion and the onset of multiple sclerosis, strengthening their association: a nested case-control study identified samples from 801 individuals who later developed multiple sclerosis. Only 35 of these individuals were EBV-seronegative and all but one seroconverted before the onset of multiple sclerosis. Furthermore, EBV seroconversion preceded the sNfL increase.^22^

We aimed to investigate the possible causative role of HHV-6A in multiple sclerosis by analysing HHV-6A serology and levels of sNfL in the prodromal phase of multiple sclerosis. The first objective of this study was to determine if HHV-6A infection before the clinical onset of multiple sclerosis was associated with higher levels of sNfL. The second objective was to determine if increasing HHV-6A seroreactivity was detectable before the increase of sNfL in the multiple sclerosis prodrome.

## Materials and methods

A nested case-control study was performed by cross-linking multiple sclerosis registries and six Swedish microbiological biobanks, as previously described in detail.^23^ Through this process, we identified and retrieved plasma or serum samples from individuals who later developed relapsing-remitting multiple sclerosis. These samples are remnants from serological testing. All included samples were collected before the clinical onset of multiple sclerosis and before the age of 40 (Supplementary Fig. 1). For each case, one control without multiple sclerosis was randomly selected and matched for biobank, sex, blood sampling date and birth date (in order of priority). The samples were categorized both according to sampling age and time to clinical onset of multiple sclerosis.

The study was performed in accordance with the Declaration of Helsinki and approved by the Regional Ethical Review Board in Umeå (2011-198-31 M with amendments 2013-226-32 M, 2017-104-32, 2017-484-32, 2018-468-32 M, 2019-03402 and 2020-00119). Participants were informed by mail with an opt-out approach. No written informed consent was required.

### Laboratory procedures

The concentration of sNfL was analysed using single molecule array (Simoa) technology and the NF-Light assay (Quanterix), as previously described.^21,24^ A bead-based multiplex assay, also previously described in detail,^8,25^ was used to detect serological responses to human herpesviruses 1–7: herpes simplex virus type 1 (HSV-1); herpes simplex virus type 2 (HSV-2); varicella zoster virus (VZV); EBV; cytomegalovirus (CMV); HHV-6A; HHV-6B; and human herpesvirus 7 (HHV-7). Seropositivity for HHV-6A was defined as a seroresponse against truncated immediate-early protein 1 from HHV-6A (IE1A).^8^ A cut-off of 50 median fluorescence intensity units (MFI) was used, as in our previous studies.^9,26^ Seropositivity for EBV was determined from the seroresponse against validated antigens and cut-offs: EBV nuclear antigen 1 (EBNA-1) truncated [amino acid (aa) 325–641] ≥ 1800 MFI; or EBNA-1 peptide (aa 385–420) ≥ 411 MFI; or viral capsid antigen p18 (aa 1–175) ≥ 2526 MFI.^9,27^

To identify samples with biochemical signs of acute disease or trauma, we analysed the concentration of C-reactive protein (CRP) with a high-sensitive multiplex immunoassay (V-PLEX Vascular Injury Panel 2 Human Kit, Mesoscale). All samples from matched cases and controls were analysed consecutively in the same batch but in random order and blinded for the technician.

The data on sNfL, CRP, HHV-6A, EBV and CMV have been published previously.^8,9,21,26,28^ In this study, we combined these data in expanded analyses to assess a different hypothesis.

### Statistical methods

#### Serostatus of HHV-6A and levels of serum neurofilament light chain

The proportions of HHV-6A-seropositive samples in matched cases and controls were compared with the McNemar test. Serum NfL was log_10_-transformed and analysed with a paired-samples *t-*test, comparing each case with its matched control. The log_10_ sNfL in HHV-6A-seropositive and -negative samples were compared with an independent samples *t-*test, stratified by case-control status.

To adjust for the natural age-dependent increase of sNfL,^29^ a within-pair ratio of sNfL was calculated for each case and its age-matched control. The ratios were log_10_-transformed and compared between HHV-6A-seropositive and -negative cases with an independent-samples *t-*test.

To further investigate the levels of sNfL in HHV-6A-seropositive and -negative samples, we plotted the within-pair ratios of sNfL against the time from serum sampling to the clinical onset of multiple sclerosis. These plots were stratified for HHV-6A serostatus and analysed with locally estimated scatterplot smoothing (loess) regression with 95% confidence intervals (CI).

As an additional approach to adjust for sampling age, we used age-adjusted *z*-scores of sNfL. These *z*-scores were calculated for each case and control using the sNfL reference application based on measurements from 4532 controls.^30^ Data regarding body mass index (BMI) at the time of serum sampling were not available and a standard BMI value of 25 was thus used in these calculations. Age-adjusted sNfL *z*-scores in HHV-6A-seropositive and -negative cases and controls were compared with a *t*-test.

The association between HHV-6A seropositivity and log_10_ sNfL was analysed with multiple linear regression, stratified by case-control status. The regression model included sampling age and serostatus for EBV. Since EBV is an age-dependent risk factor for multiple sclerosis,^9^ these analyses were stratified by median sampling age. The model was also adjusted for sex, indirectly associated with sNfL through the risk of trauma.

To further assess the association between HHV-6A serostatus and elevated sNfL, a logistic regression was performed, calculating the odds ratio (OR) with 95% CI for HHV-6A as a risk factor for elevated sNfL. Elevated sNfL was defined as age-adjusted sNfL *z*-score >2. The model also included serostatus of EBV and sex. These analyses were stratified by case-control status and median sampling age.

To determine if combined HHV-6A and EBV seropositivity affected sNfL levels, we performed a *t-*test of log_10_ sNfL in cases with singular versus combined seropositivity for HHV-6A and EBV. Sampling age was accounted for by analysing age-adjusted sNfL *z*-scores.

#### Time relation of HHV-6A antibodies and serum neurofilament light chain

The included samples were donated up to 33 years (median 10.4 years) before the clinical onset of multiple sclerosis. Together with the matched samples, this material reflects a wide time span, enabling temporal analysis of the order of events on the group level.

The natural variation of sNfL is too large to decide if an individual sample was drawn during the multiple sclerosis prodrome. However, by analysing many samples from a wide time range, the transient shift towards increasing levels of sNfL is revealed.^21^ Events occurring before the increasing sNfL levels are likely occurring before the subclinical onset of multiple sclerosis.

To assess if increasing HHV-6A seroreactivity was detectable before the rise of sNfL, we calculated the within-pair ratio of HHV-6A seroreactivity (measured as MFI) for each matched case-control pair. To reduce the effect of the random technical noise in the seronegative samples, and avoid division by zero, these plots were calculated with a limit of detection (LOD) of 50 MFI. These ratios were plotted against the time from the serum sampling to the clinical onset of multiple sclerosis and analysed using loess regression with 95% CI. This plot was then compared with the previously reported loess regression of within-pair ratio of sNfL against the time to the clinical onset of multiple sclerosis.^21^ For comparison, we also calculated loess regressions of within-pair MFI ratios for herpesviruses HSV-1, HSV-2, VZV, CMV, HHV-6B and HHV-7, plotted against the time to the clinical onset of multiple sclerosis. These plots were also calculated with a LOD of 50 MFI.

#### Sensitivity analyses

Elevated levels of NfL have been reported in many other conditions, such as traumatic brain injury and CNS infections.^31,32^ To exclude individuals with biochemical signs of acute disease or trauma, sensitivity analyses were performed in samples with CRP <5 mg/l.

The method to calculate age-adjusted sNfL *z*-scores is currently not applicable to children below the age of 17, for whom (*n* = 91 of 1038) the *z*-scores were calculated as if these individuals were 17 years old. Sensitivity analyses were performed to account for the uncertainty of these *z*-scores, excluding samples drawn before the age of 17.

A few samples were collected closely before the clinical onset of multiple sclerosis (Supplementary Fig. 1), conferring uncertainty about whether the samples were truly presymptomatically collected. A set of sensitivity analyses were thus performed limited to samples collected >1–4 years before the clinical onset.

Since seropositivity against EBV appears to be necessary for developing multiple sclerosis, the prodromal phase may also be dependent on EBV. Additional sensitivity analyses were thus limited to EBV seropositive samples.

Statistical tests were two-tailed. The significance level was 0.05. Statistical analyses were performed in R, version 4.2.1, and SPSS, version 28. All graphs were constructed in R.

## Results

We identified and retrieved samples from a total of 670 matched case-control pairs. Owing to insufficient sample volume, 151 sets were excluded, leaving 519 matched case-control pairs for the final analyses. The median age at sampling was 24.7 years and the median time to clinical onset of multiple sclerosis was 9.5 years. Demographic data have been described previously.^21^

### Serostatus of HHV-6A and levels of serum neurofilament light chain

The proportion of HHV-6A-seropositive samples was significantly higher in cases than in controls (Table 1). A significantly higher level of sNfL was observed in cases compared to age-matched controls. Comparing HHV-6A-seropositive and -negative cases, the levels of sNfL were significantly higher in the seropositive cases. Similar results were observed for age-adjusted values, although not statistically significant (Table 1).

**Table 1:** Serostatus of HHV-6A and sNfL in cases and controls

	Cases			Controls		*P*
HHV-6A+ (*n*, %)	208/519, 40%			132/519, 25%		<0.001^a^
sNfL (geometric mean, pg/ml)	7.07 (6.70–7.45)			6.21 (5.93–6.50)		<0.001^b^
	HHV-6A+	HHV-6A−	*P*	HHV-6A+	HHV-6A−	*P*
sNfL (geometric mean, pg/ml)	7.65 (6.99–8.37)	6.70 (6.28–7.15)	0.02^c^	6.35 (5.82–6.93)	6.16 (5.84–6.50)	0.57^c^
sNfL ratio (geometric mean)	1.21 (1.09–1.34)	1.09 (1.02–1.17)	0.11^c^	0.88 (0.78–1.00)	0.88 (0.82–0.94)	0.92^c^
Age-adjusted sNfL *z*-score (mean)	1.12 (0.96–1.28)	0.92 (0.79–1.06)	0.07^c^	0.76 (0.55–0.98)	0.73 (0.61–0.85)	0.79^c^

Mean values with 95% confidence intervals. The sNfL ratio was calculated for each matched case-control set. Age-adjusted sNfL *z*-scores were calculated with sNfL reference application. HHV-6A seropositivity was defined as a seroresponse >50 MFI against HHV-6A antigen IE1A. HHV-6A = human herpesvirus 6A; MFI = median fluorescence intensity; sNfL = serum neurofilament light chain.

^a^McNemar test.

^b^Paired samples *t*-test.

^c^Independent samples *t*-test.

**Figure 1:**
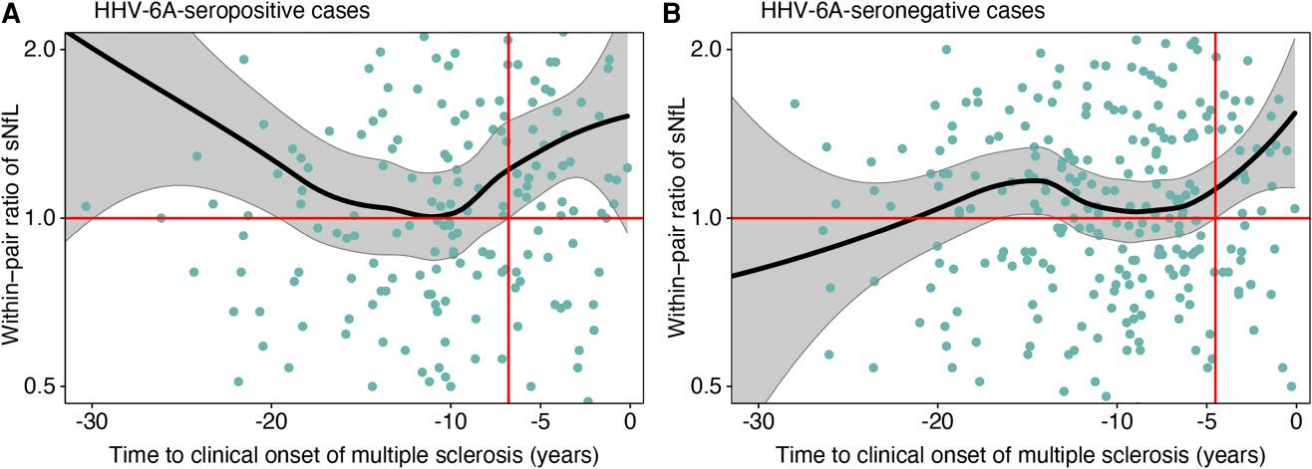
**Locally estimated scatterplot smoothing (loess) regression with 95% confidence intervals for within-pair sNfL ratio against time to onset of multiple sclerosis**. (**A**) HHV-6A-seropositive cases. (**B**) HHV-6A-seronegative cases. Within-pair serum neurofilament light chain (sNfL) ratio was calculated for each matched case-control pair. Matched controls were included regardless of their HHV-6A serostatus. The time to onset of multiple sclerosis was calculated as the interval from the sampling date to the date of the first symptom indicative of multiple sclerosis. The time points for significant within-pair ratios of MFI (95% CI > 1.0) are marked with vertical red lines. Log_2_ scaled *y*-axis, limited to a ratio of 2 in either direction. HHV-6A seropositivity was defined as a seroresponse >50 MFI against HHV-6A antigen IE1A. HHV-6A = human herpesvirus 6A.

The level of sNfL was significantly higher in HHV-6A-seropositive cases than in their matched controls from more than 6 years prior to the clinical onset of multiple sclerosis (Fig. 1A). However, a similar increase of sNfL was also observed in HHV-6A-seronegative cases, where sNfL was significantly higher than in their matched controls from more than 4 years before the clinical onset (Fig. 1B). The confidence intervals were overlapping and the loess regressions of these two groups were not significantly different.

We observed a significant association between HHV-6A seropositivity and higher sNfL among cases, but not in controls (Table 2). The association between HHV-6A and sNfL was driven by findings in samples drawn before the median age of 24.7 years.

**Table 2:** Linear regressions of sNfL levels in cases and controls

	Cases			Controls		
	sNfL level	95% CI	*P*	sNfL level	95% CI	*P*
**All sampling ages**		***n* = 519**			***n* = 519**	
HHV-6A+	+11%	+0.2–24%	0.045	+1%	−9–12%	0.84
EBV+	−6%	−24–16%	0.56	−4%	−19–14%	0.65
Male sex	+26%	+10–45%	<0.001	+20%	+7–35%	0.002
Age at sampling, per year	+2%	+1–3%	<0.001	+2%	+2–3%	<0.001
**Sampling age below median (<24.7)**		***n* = 259**			***n* = 259**	
HHV-6A+	+24%	+6–45%	0.007	+5%	−9–20%	0.50
EBV+	±0%	−22–29%	0.99	−3%	−21–19%	0.77
Male sex	+26%	+4–52%	0.02	+12%	−3–30%	0.13
Age at sampling, per year	±0%	−2–2%	0.73	−1%	−2–1%	0.36
**Sampling age above median (**≥**24.7)**		***n* = 260**			***n* = 260**	
HHV-6A+	+1%	−13–16%	0.92	−1%	−14–14%	0.88
EBV+	−3%	−46–75%	0.93	+12%	−16–51%	0.44
Male sex	+25%	+2–52%	0.03	+27%	+6–52%	0.01
Age at sampling, per year	+3%	+1–5%	<0.001	+5%	+3–7%	<0.001

Multiple linear regression with Log_10_ sNfL as dependent variable. The estimates of sNfL levels are reported as percentage change, e.g. in cases, seropositivity for HHV-6A was associated with an 11% higher level of sNfL. CI = confidence interval; EBV+ = seropositive for Epstein-Barr virus; HHV-6A+ = seropositive for human herpesvirus 6A; sNfL = serum neurofilament light chain.

Elevated sNfL (age-adjusted sNfL *z*-score > 2) was observed in 104 cases and 73 controls (*P* = 0.008). In young cases (sampling age < median of 24.7 years), seropositivity for HHV-6A was significantly associated with a higher risk of elevated sNfL; OR = 1.91 (95% CI 1.04–3.52), *P* = 0.04. No such association was observed in controls (Supplementary Table 1).

The level of sNfL was significantly higher in cases with combined seropositivity for HHV-6A and EBV, compared to those only seropositive for EBV (geometric mean 7.67 versus 6.76, *P* = 0.03). Similar findings were observed when accounting for age, although not statistically significant; mean age-adjusted sNfL *z*-score 1.10 versus 0.89, *P* = 0.06.

Only eight cases were seropositive against HHV-6A and simultaneously EBV seronegative, limiting further interaction analyses. Levels of sNfL in categories with different combinations of EBV and HHV-6A serostatus are presented in Supplementary Table 2.

### Time relation of HHV-6A antibodies and serum neurofilament light chain

**Figure 2:**
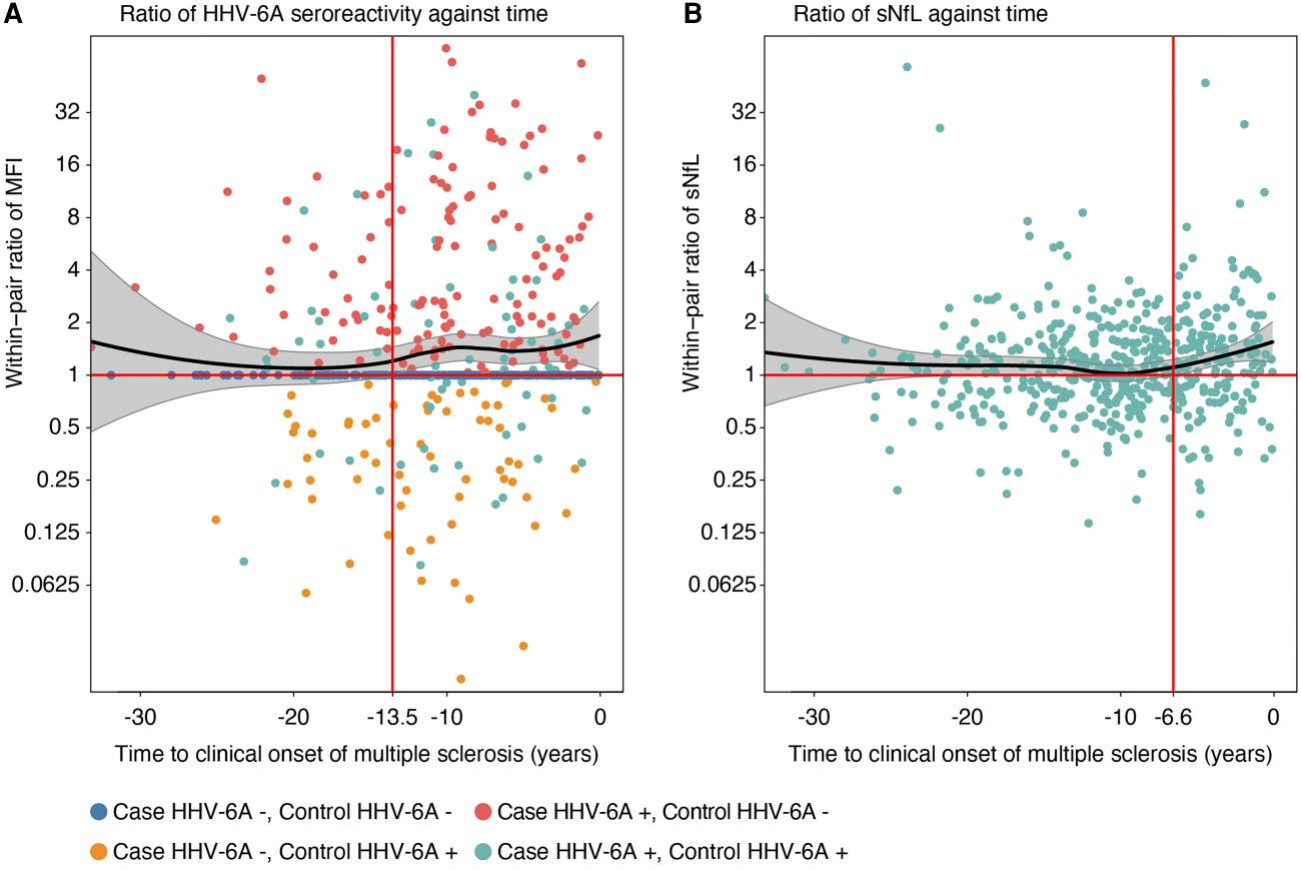
**Time relation of HHV-6A antibodies and sNfL**. (**A**) Loess regression with 95% confidence intervals for within-pair ratio of seroreactivity (MFI) for HHV-6A antigen IE1A against time to clinical onset of multiple sclerosis. (**B**) Loess regression of within-pair serum neurofilament light chain (sNfL) ratio against time to onset of multiple sclerosis, as previously reported.^21^ Within-pair ratios were calculated for each case and matched control and a cut-off of 50 MFI. The time to onset of multiple sclerosis was calculated as the interval from the sampling date to the date of the first symptom indicative of multiple sclerosis. The time-points for significant within-pair ratios (95% CI > 1.0) are marked with vertical lines. HHV-6A = human herpesvirus 6A.

The within-pair ratios of HHV-6A seroreactivity and sNfL were plotted against the time to the clinical onset and analysed with loess regression. As visualized by the 95% CI of the loess regression, a statistically significant ratio of HHV-6A seroreactivity was detectable from >13.5 years before the clinical onset of multiple sclerosis (Fig. 2A). As previously reported, the sNfL ratio increased from 10 years before the clinical onset.^21^ A significant sNfL ratio was observed from 6.6 years before the clinical onset (Fig. 2B). The increasing HHV-6A seroreactivity was thus detectable before the rise of sNfL in samples from individuals who later developed multiple sclerosis.

For comparison, the ratios of seroreactivities for human herpesviruses HSV-1, HSV-2, VZV, CMV, HHV-6B and HHV-7 were also plotted. No significant increases in MFI ratios were observed for these viruses (Supplementary Fig. 2).

### Sensitivity analyses

To identify and exclude samples drawn at acute disease, we used available CRP estimates.^28^ We selected those with CRP <5 mg/l (300 cases and 310 controls) for these sensitivity analyses. The level of sNfL remained significantly higher in HHV-6A-seropositive cases than in negative cases (geometric mean 7.91 versus 6.68, *P* = 0.01). The within-pair ratio of sNfL in HHV-6A-seropositive and -negative cases (geometric mean ratio 1.29 versus 1.10, *P* = 0.12) were similar to ratios for all samples. This was calculated for pairs where both the case and the control had CRP <5 mg/ml (*n* = 191). The association between HHV-6A and higher levels of sNfL remained similar in all sampling ages (+14%, 95% CI 0–29%, *P* = 0.06) and still significant in the younger group (+26%, 95% CI +4–52%, *P* = 0.02). Cases with combined seropositivity for HHV-6A and EBV still had significantly higher levels of sNfL compared to those only seropositive for EBV (geometric mean 7.99 versus 6.69, *P* = 0.01).

In the sensitivity analyses of age-adjusted sNfL *z*-scores, we also excluded samples drawn before the age of 17. Still, the results remained similar. The sNfL *z*-scores were significantly higher in HHV-6A-seropositive than in -negative cases (mean *z*-score 1.20 versus 0.91, *P* = 0.045). Cases with combined seropositivity for HHV-6A and EBV had significantly higher sNfL *z*-scores than those only seropositive for EBV (mean *z*-score 1.19 versus 0.90, *P* = 0.04). The logistic regression, assessing HHV-6A as a risk factor for elevated sNfL, also remained similar in cases below median age (OR = 2.19, 95% CI 0.92–5.23, *P* = 0.08).

Sensitivity analyses of samples drawn >1–4 years before the clinical onset of multiple sclerosis yielded similar results (Supplementary Table 3).

Finally, highly similar results were also observed when excluding EBV seronegative samples. As in the primary analysis, HHV-6A seropositivity was significantly associated with the level of sNfL in the younger half of the cases, but not among the older cases (Supplementary Table 4).

## Discussion

In this study, we investigated the relationship between HHV-6A serostatus and sNfL levels in samples collected many years prior to the clinical onset of multiple sclerosis.

In the linear regression analysis, HHV-6A seropositivity was significantly associated with higher levels of sNfL, also when adjusted for sampling age, EBV serostatus and sex. The association was only present in samples collected from cases at a young (below median, 24.7 years) age, where HHV-6A seropositivity was associated with a doubled risk of elevated sNfL. This finding may reflect that this is the age when HHV-6A may contribute to the multiple sclerosis prodrome. The level of sNfL was also higher in cases with combined seropositivity for HHV-6A and EBV compared to those only seropositive for EBV. Similar, although not statistically significant, results were observed when analysing mean age-adjusted sNfL *z*-scores and within-pair sNfL ratios. The results also remained similar in sensitivity analyses. No corresponding associations between HHV-6A and sNfL were observed in the control group.

Using presymptomatically collected serum samples, the current study design minimizes the risk of reverse causation. Spanning from early childhood to adulthood, the collection of samples provides a unique opportunity to assess the order of events in the pathogenesis of multiple sclerosis. Increasing seroreactivity against HHV-6A was detectable in cases many years before the increase of sNfL, suggesting a time relation where HHV-6A antibodies precede demyelination and axonal injury. This was not part of a non-specific increase of antibodies belonging to a subclinical multiple sclerosis pathological process, as similar findings were not found for six other human herpesviruses (Supplementary Fig. 2).

This study also entails limitations that need to be addressed. The conversion to age-adjusted sNfL *z*-scores resulted in a mean sNfL *z*-score of 0.74 in the control samples, which is higher than expected. One possible reason would be that many samples were initially collected to diagnose acute disease, which may affect the levels of sNfL. Unfortunately, no clinical data at the time of serum sampling were available for cases or controls. However, since sensitivity analyses, excluding samples with CRP ≥5 mg/l, yielded highly similar results, this may not be the sole explanation. Other factors, such as evaporation, may also affect the levels of sNfL. Still, this would affect samples from cases and controls equally and would not affect the validity of our findings. Furthermore, we did not have access to height or weight data at the time of serum sampling. The level of sNfL decreases with higher BMI, likely by dilution in the larger circulating blood volume.^33^ Adolescent overweight is a recognized risk factor for multiple sclerosis.^34,35^ It can thus be assumed that the cases had a higher mean BMI than controls, which confers the risk of introducing a systematic bias. However, such a bias would lead to an underestimation of the sNfL *z*-scores in cases, resulting in a false negative result. This limitation should thus not question the validity of the results. In addition, the levels of sNfL might also be affected by differences between the cohorts and the laboratory methods in the current study and the sNfL reference database.

In the plots and loess regressions, the age-dependent variation of sNfL is accounted for by the use of the within-pair ratio, providing age-matched comparisons. Using the same approach for seroreactivity, which is not necessarily age-dependant, might seem unjustified. However, this approach enables plotting seroreactivity against the time to multiple sclerosis onset, simultaneously providing comparisons with controls matched for biobank, sex and age. Furthermore, this method enables comparisons of the time points for increasing seroreactivity and sNfL levels.

The antigens and MFI cut-offs for determining serostatus of HSV-1, HSV-2, VZV, EBV and CMV have all been validated against established reference assays.^27^ However, this is not the case for HHV-6A, for which no reliable reference assay is yet available. The assay has only been partly validated for specificity in sera from children with exanthema subitum (also known as roseola infantum or sixth disease), which is caused by HHV-6B.^36^ The validation indicated negligent cross-reactivity for IE1A regarding HHV-6A and -6B.^8^ Since no differences between cases and controls were observed for HHV-6B seroreactivity,^9^ the association between HHV-6B and sNfL was not assessed here.

Furthermore, no validated cut-off has been established for IE1A. In consistence with our previous studies, we defined HHV-6A seropositivity as a seroresponse against IE1A > 50 MFI.^9,26^ Other studies have used the 75th percentile of IE1A MFI in controls as the cut-off.^8,37^ In the current sample, that value corresponds to an MFI of 50.7. The different definitions of serostatus would thus yield highly similar or identical results. Furthermore, the cut-off used in the current study has previously been tested in sensitivity analyses and shown to be robust.^9^ Still, the method to differentiate HHV-6A serostatus is less certain than the approach for EBV serostatus. This is a limitation, as in all current studies of HHV-6A serology.

Finally, it remains possible that the association between HHV-6A and multiple sclerosis represents an epiphenomenon due to viral reactivation in injured oligodendrocytes. However, the observation that increasing HHV-6A antibodies were detectable before increasing sNfL argues against such a mechanism, which would likely result in the reversed time-order.

Several hypotheses for HHV-6A in the pathogenesis of multiple sclerosis have been suggested.^5,38,39^ A direct injury from lytic HHV-6A infection in oligodendrocytes could cause cell death, demyelination and subsequent inflammation. Similar processes have been observed for JC Polyomavirus in progressive multifocal leukoencephalopathy and measles virus in subacute sclerosing panencephalitis.^38^ It has also been suggested that the pathogenic effect could be caused by molecular mimicry.^38^ This concept implies that viral proteins share amino acid sequences or structures with host cell proteins. These viral antigens are thereby suggested to activate autoreactive immune-competent cells, causing aberrant inflammation and cell damage. For EBV, two epitopes of EBNA-1 display amino acid homology with CNS host antigens, namely anoctamin 2 (ANO2)^40^ and GlialCAM.^41^ Carriage of ANO2 antibodies associate with an increased risk of multiple sclerosis, highly suggestive of a pathogenic role.^40^ A homologous amino acid sequence with potential for molecular mimicry has also been described for an HHV-6 peptide and myelin basic protein, a putative autoantigen for multiple sclerosis.^42^ An alternative mechanism of virus-induced autoimmunity has also been suggested: during viral replication, the envelope is formed from the host cell membrane and can thus incorporate lipids and proteins from the host.^43^ As a neurotrophic virus, the envelope of HHV-6A may contain proteins and lipids from oligodendrocytes, which could trigger autoimmune reactions directed against these cells.^44^

However, the previous observations of total EBV seroprevalence in adults with multiple sclerosis suggest that EBV is a prerequisite, but insufficient cause of the disease.^3,4^ Neither do the results of the current study indicate that HHV-6A is the sole cause of multiple sclerosis, instead providing an increased risk. The level of sNfL increased significantly before the clinical onset of multiple sclerosis in both HHV-6A-seropositive and -negative cases (Fig. 1A and B), indicating that HHV-6A is no prerequisite for multiple sclerosis but rather a contributing factor in a proportion of cases. Only 8 of 519 samples from individuals who later developed multiple sclerosis were seropositive against HHV-6A and simultaneously seronegative against EBV. All these samples were drawn more than 10 years before the clinical onset of multiple sclerosis, leaving ample time for late EBV infection. Isolated HHV-6A infection is thus rare during the presymptomatic phase of multiple sclerosis and virtually non-existent when the disease is fully developed.^3,4^ Instead, the observations in the current study support the hypothesis that both EBV and HHV-6A contribute to the development of multiple sclerosis.^39^ In consistence, HHV-6A, but not HHV-6B, co-infect B cells latently infected with EBV, resulting in a subsequent increase in the expression of EBV antigens.^45-47^ Furthermore, additive interactions have been observed between EBV and HHV-6A seropositivity regarding the risk of multiple sclerosis.^8^ Infection from EBV during early childhood is typically mild or asymptomatic, while infection at a later age often causes infectious mononucleosis. The latter is highly associated with the risk of multiple sclerosis. Late EBV infection, causing infectious mononucleosis, might thus be sufficient to cause multiple sclerosis in individuals with genetic susceptibility and/or other risk factors. In contrast, early EBV infection could require a second hit by HHV-6A infection to reactivate latent EBV and initiate the inflammatory cascade leading to multiple sclerosis. This hypothesis is consistent with the observations in the present study, where increasing seroreactivity against HHV-6A was detectable years before the rising levels of sNfL. This indicates that elevated HHV-6A antibodies precede the immune-mediated axonal injury in a subset of individuals that will develop multiple sclerosis. However, the current study can merely indicate the order of events at the group level. While the large number of samples and the many years observed between increasing HHV-6A seroreactivity and sNfL support the relevance of these observations, the lack of serial samples limits the possibility to suggest causality. Testing the suggested hypothesis requires an extensive longitudinal study of presymptomatically collected serial samples, identifying the time points for both EBV and HHV-6A seroconversion. Furthermore, there is a need for PCR studies of HHV-6A and -6B excretion in several clinical materials, including saliva and/or white blood cells, combined with type-specific HHV-6 serology.

In conclusion, we report a significant association between HHV-6A seropositivity and the level of sNfL in samples from individuals who later developed multiple sclerosis, likely reflecting the prodromal phase of the disease. In addition, increasing seroreactivity against HHV-6A was detectable before the rise of sNfL. These findings support the hypothesis that HHV-6A may contribute to the pathogenesis of multiple sclerosis in a proportion of cases, but do not show that HHV-6A infection is essential for disease development.

## Supplementary Material

awad374_Supplementary_Data

## Data Availability

The data that support the findings of this study are available from the corresponding author upon reasonable request.
