# Supplementary material for: Human herpesvirus 6A and axonal injury before the clinical onset of multiple sclerosis
Source: Brain. 2023 Oct 31;147(1):177–85. doi: 10.1093/brain/awad374 (PMC10766246; doi:10.1093/brain/awad374)

Supplements

Supplementary Table 1. Serostatus for HHV-6A and risk of elevated sNfL *z*-score

	Cases			Controls		
**All sampling ages**	**OR**	**95 % CI**	***P***	**OR**	**95 % CI**	***P***
HHV-6A +	1.21	(0.78–1.88)	0.39	1.11	(0.63–1.96)	0.71
EBV +	0.62	(0.29–1.34)	0.22	0.95	(0.39–2.31)	0.91
Male sex	1.19	(0.69–2.05)	0.54	2.30	(1.29–4.08)	0.005
**Sampling age below median (<24.7)**		**(n = 259)**			**(n = 259)**	
HHV-6A +	1.91	(1.04–3.52)	0.04	1.69	(0.78–3.65)	0.18
EBV +	0.58	(0.25–1.36)	0.21	0.64	(0.23–1.79)	0.40
Male sex	1.11	(0.53–2.33)	0.77	2.23	(1.03–4.84)	0.04
**Sampling age above median (≥24.7)**		**(n = 260)**			**(n = 260)**	
HHV-6A +	0.73	(0.38–1.40)	0.35	0.73	(0.31–1.70)	0.46
EBV +	0.92	(0.09–9.68)	0.94	2.55	(0.31–20.93)	0.38
Male sex	1.30	(0.56–3.00)	0.55	2.47	(1.04–5.87)	0.04

Elevated sNfL was defined as age-adjusted sNfL z-score >2; sNfL, serum neurofilament light chain; OR, odds ratio; CI, confidence interval; HHV-6A, Human herpesvirus 6A; EBV, Epstein-Barr virus.

Supplementary Table 2. Levels of sNfL in categories of combined EBV and HHV-6A serostatus

**Serostatus**	**Cases**	**Controls**	***P***
HHV-6A +	208 (40%)	132 (25%)	<0.001
sNfL (pg/mL)	7.65	6.35	0.006
EBV +	483 (93%)	479 (92%)	0.63
sNfL (pg/mL)	7.12	6.21	<0.001
**Combined serostatus:**			
HHV-6A - EBV -	28 (5%)	35 (7%)	0.36
sNfL (pg/mL)	6.15	6.42	0.68
HHV-6A - EBV +	283 (55%)	352 (68%)	<0.001
sNfL (pg/mL)	6.76	6.14	0.03
HHV-6A + EBV -	8 (2%)	5 (1%)	0.40
sNfL (pg/mL)	7.16	5.05	0.13
HHV-6A + EBV +	200 (39%)	127 (24%)	<0.001
sNfL (pg/mL)	7.67	6.41	0.01

Proportions are reported as percentages and sNfL as geometric mean. HHV-6A seropositivity was defined as a seroresponse >50 MFI against HHV-6A antigen IE1A. EBV seropositivity was determined from the following antigens and cut-offs: EBV nuclear antigen 1 (EBNA-1) truncated ≥1800 MFI; or EBNA-1 peptide ≥411 MFI; or viral capsid antigen p18 ≥2526. HHV-6A, human herpesvirus 6A; EBV, Epstein-Barr virus; MFI, median fluorescence intensity.

Supplementary Table 3. Sensitivity analyses of samples drawn > 1–4 years before the clinical onset of multiple sclerosis

Sample collection	Serostatus	sNfL level	95% CI	*P*
> 1 year before clinical onset	HHV-6A + *	+13%	+2% – +26%,	0.02
> 2 years before clinical onset	HHV-6A + *	+14%	+2% – +26%,	0.02
> 3 years before clinical onset	HHV-6A + *	+14%	+2% – +26%,	0.02
> 4 years before clinical onset	HHV-6A + *	+11%	-1% – +24%,	0.08

*All sampling ages, adjusted for EBV serostatus, male sex and age at sampling.

Multiple linear regression with Log_{10} sNfL as dependent variable. The estimates of sNfL levels are reported as percentage change, e.g., in samples collected > 1 year before clinical onset, seropositivity for HHV-6A was associated with a 13% higher level of sNfL. sNfL, serum neurofilament light chain, CI, confidence interval, HHV-6A +, seropositive for human herpesvirus 6A.

Supplementary Table 4. Linear regressions of serum NfL levels in EBV seropositive cases and controls

	Cases (n = 483)			Controls (n = 479)		
**All sampling ages**	**sNfL level**	**95% CI**	***P***	**sNfL level**	**95% CI**	***P***
HHV-6A +	+11%	(-1%–24%)	0.07	+3%	(-8%–14%)	0.65
Male sex	+27%	(+10%–47%)	0.001	+23%	(+8%–40%)	0.001
Age at sampling, per year	+2%	(+1%–3%)	<0.001	+2%	(+2%–3%)	<0.001
**Sampling age below median (<24.7)**		**(n = 227)**			**(n = 233)**	
HHV-6A +	+24%	(+4%–47%)	0.02	5%	(-9%–22%)	0.47
Male sex	+25%	(+0.5%–55%)	0.045	14%	(-3%–35%)	0.11
Age at sampling, per year	±0%	(-2%–3%)	0.77	-1%	(-3%–1%)	0.23
**Sampling age above median (≥24.7)**		**(n = 256)**			**(n = 246)**	
HHV-6A +	+1%	(-13%–17%)	0.91	±0%	(-14%–16%)	0.96
Male sex	+26%	(+3%–55%)	0.02	+30%	(+7%–57%)	0.008
Age at sampling, per year	+3%	(+1%–5%)	0.001	+5%	(+3%–7%)	<0.001

Multiple linear regression with Log_{10} sNfL as dependent variable. The estimates of sNfL levels are reported as percentage change, e.g., in cases, seropositivity for HHV-6A was associated with an 11% higher level of sNfL. sNfL, serum neurofilament light chain, CI, confidence interval, HHV-6A +, seropositive for human herpesvirus 6A.

Supplementary Figure 1. Age at sampling and clinical onset of multiple sclerosis

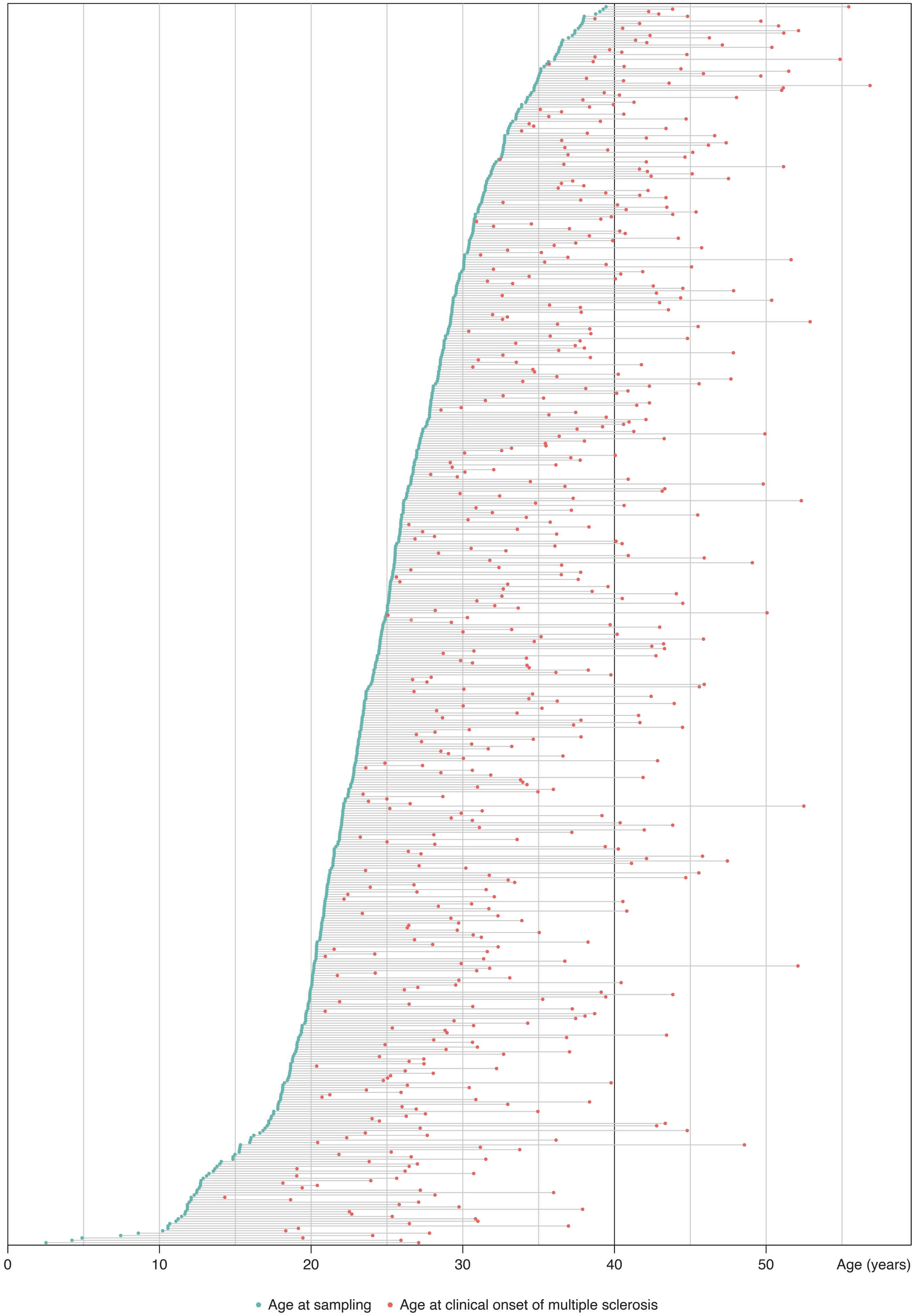

Supplementary Figure 2. Seroresponse for herpesviruses against time to clinical onset of multiple sclerosis

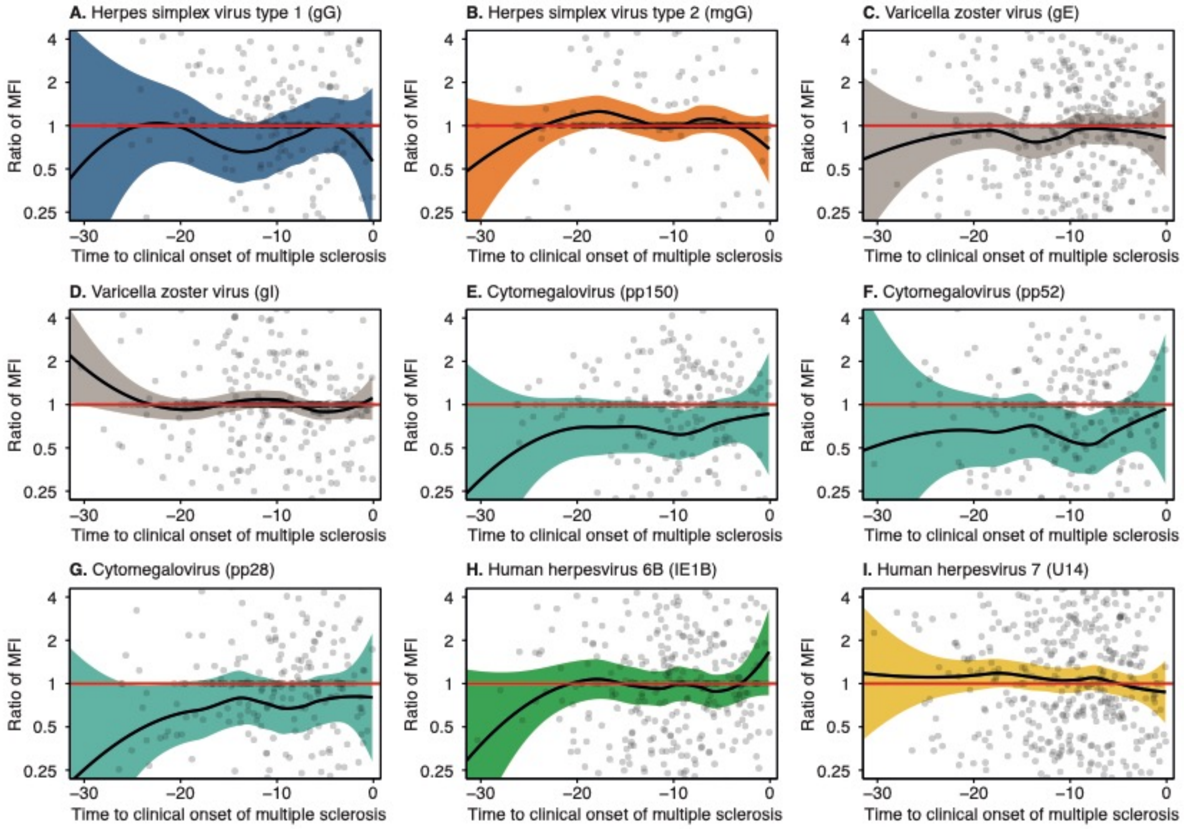

Loess regression with 95% confidence intervals (coloured fields) for within-pair ratio of seroreactivity (MFI) of herpesviruses 1–3 and 5–7. Within-pair ratios were calculated for each case and matched control. To reduce the effect of the technical noise and avoid division by zero, a limit of detection of 50 MFI was used in the calculation. The time to onset of multiple sclerosis was calculated as the interval from the sampling date to the date of the first symptom indicative of multiple sclerosis. Log_2 scaled y-axis, limited to a ratio of 4 in either direction. MFI, median fluorescence intensity; loess, locally estimated scatterplot smoothing.

Supplementary Figure 3. Scattered boxplot of sNfL in cases and controls

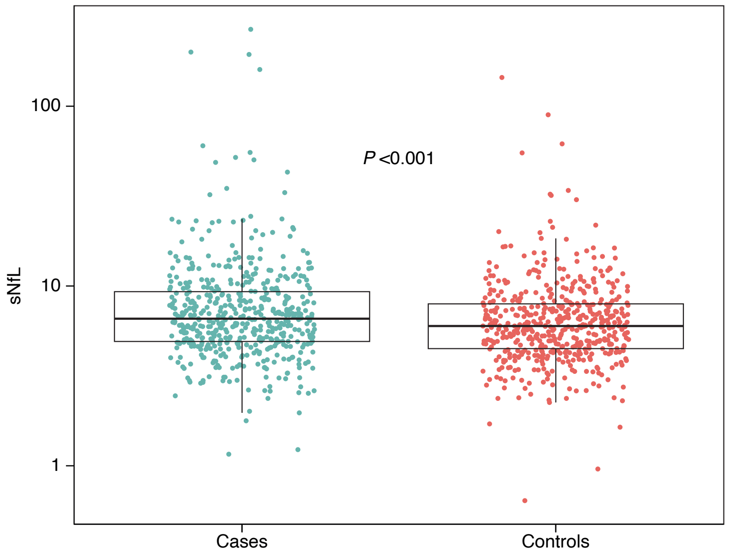

Supplement: awad374_Supplementary_Data [file awad374_supplementary_data.pdf]
